# Enhanced Resolution in EPR Spectroscopy Using *para*‐Hydrogen Matrices

**DOI:** 10.1002/anie.202518517

**Published:** 2025-11-19

**Authors:** Adrián Portela‐González, Wolfram Sander, André K. Eckhardt

**Affiliations:** ^1^ Lehrstuhl für Organische Chemie II Ruhr‐Universität Bochum Universitätsstraße 150 44801 Bochum Germany

**Keywords:** High‐resolution EPR spectroscopy, Matrix isolation, Phosphorus, Photolysis, Radicals

## Abstract

We developed a matrix isolation experiment utilizing a closed‐cycle helium cryostat, which operates at 2.5 K and enables electron paramagnetic resonance (EPR) measurements in solid *para*‐hydrogen (*p*‐H_2_) matrices. The EPR spectra of the persistent 2,2,6,6‐tetramethylpiperidinyloxyl (**TEMPO**) radical and an in situ generated *P*‐centered mono‐radical were recorded at 2.5 K in *p*‐H_2_. The spectrum of **TEMPO** in *p*‐H_2_ shows a narrower linewidth compared to argon, and its matrix‐isolated spectrum and simulation are reported. The *P*‐centered mono‐radical was generated in *p*‐H_2_ via in situ photolysis of the corresponding phosphorus iodide, which affords roughly a three‐fold increase in spectral resolution compared to argon, as well as higher sensitivity due to the non‐existing cage effect in soft *p*‐H_2_.

## Graphical abstract

We demonstrate the advantageous properties of solid *para*‐hydrogen (*p*‐H_2_) matrices for electron paramagnetic resonance (EPR) spectroscopy by investigating the 2,2,6,6‐tetramethylpiperidinyloxyl (**TEMPO**) radical and an in situ generated *P*‐centered mono‐radical. The spectral resolution of the *P*‐centered radical in *p*‐H_2_ is roughly enhanced by a factor of three compared to argon.

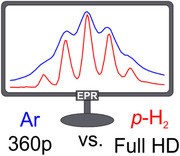

Electron paramagnetic resonance (EPR) is a powerful technique that permits the study of the electronic structure of species with unpaired electrons. This technique finds application in a variety of areas, including biochemistry,^[^ ^1^ ^]^ biophysics,^[^ ^2^ ^]^ material sciences,^[^ ^3^ ^]^ energy storage,^[^ ^4^ ^]^ inorganic chemistry,^[^ ^5^ ^]^ physical chemistry,^[^ ^6^ ^]^ physical organic chemistry,^[^ ^7^ ^]^ catalysis,^[^ ^8^ ^]^ astrochemistry,^[^ ^9^ ^]^ and even emerging fields such as quantum computing.^[^ ^10^ ^]^ The parameters obtained by EPR spectroscopy depend on several factors, such as sample preparation and concentration, thus complicating its interpretation and comparison with theoretical predictions. Usually, EPR spectroscopy experiments are performed in the condensed phase, which affects the spectrum of the paramagnetic species studied due to solvent‐solute (among other) interactions. On the other hand, the matrix isolation technique affords spectroscopic data of isolated molecules nearly unaffected by intermolecular interactions. Matrix isolation EPR spectroscopy of mono‐radicals provides anisotropic spectra in the rigid limit, from which the interactions of the spin with the different magnetically active nuclei can be extracted. The only restriction is the spectral resolution. Acquiring accurate physical parameters is not only relevant for spectroscopic characterization but also for benchmarking of EPR theoretical predictions, currently lacking non‐interactive accurate experimental data.^[^ ^11^, ^12^ ^]^ High‐resolution matrix spectroscopic data have been obtained with infrared (IR) spectroscopy using *para*‐hydrogen (*p*‐H_2_) as the host material.^[^ ^13^ ^]^

*p*‐H_2_ is the energetically preferred nuclear spin isomer of hydrogen in which its nuclear spins are antisymmetric, affording a singlet state (*I* = 0). This contrasts with its other isomer, *ortho*‐hydrogen (*o*‐H_2_), where both spins are symmetric, leading to a triplet state (*I* = 1). At room temperature, the populations of both isomers are equal, leading to normal‐hydrogen (*n*‐H_2_) with a 3:1 ratio in favor of the *ortho* isomer.^[^ ^14^ ^]^ *p*‐H_2_ has a spherical symmetry and, therefore, a multipole moment equal to zero, whereas *o*‐H_2_ has a quadrupole magnetic moment. This conceives *p*‐H_2_, to some extent, with similar properties to those of noble gases regarding its interaction with the guest molecules for matrix isolation purposes.^[^ ^14^ ^]^ Moreover, *p*‐H_2_ exhibits a hexagonal‐closed pack (hcp) crystalline packing, whereas most noble gases often present more than one crystalline structure, thus leading to different matrix sites and spectral broadening. The crystalline packing of solid *p*‐H_2_ shows a large lattice constant,^[^ ^15^ ^]^ which additionally makes *p*‐H_2_ a soft matrix material. Due to these properties, *p*‐H_2_ is described as a quantum solid.

The main limitation for performing studies with solid *p*‐H_2_ relates to the low temperatures (<4.0 K) required to prepare and maintain these matrices stable during the experiment. The wider availability of closed‐cycle helium cryostats operating at 3 K have facilitated the use of *p*‐H_2_ as a matrix material, which is nowadays common for matrix isolation IR studies resulting in high‐resolution spectra.^[^ ^16^, ^17^ ^]^ In some cases, the resolution of spectra recorded in the gas phase is exceeded.^[^ ^18^ ^]^ The softness of solid *p*‐H_2_ together with its small molecular size prevents this matrix material from suffering the cage effect, which is usually present in rigid noble gas matrices.^[^ ^13^ ^]^ Typically, in photolysis experiments, fragments remain within the same matrix cage, favoring radical–radical recombination reactions. Therefore, the photolysis yields in *p*‐H_2_ are consistently higher than in other matrices due to the diminished cage effect.^[^ ^19^ ^]^ To the best of our knowledge, matrix isolation EPR experimental setups operating below 5 K do not exist so far. Reasons might be technical difficulties, including the magnetic field or space limitations due to the small resonance cavity of the EPR spectrometer. Therefore, the available EPR data in *p*‐H_2_ in the literature is very scarce and reduced to very small radicals such as hydrogen^[^ ^20^, ^21^, ^22^ ^]^ and deuterium,^[^ ^22^ ^]^ as well as methyl radicals^[^ ^23^ ^]^ condensed into a plate cooled by liquid helium.

Here we present our new matrix isolation continuous wave (CW) EPR experimental setup with a powerful closed‐cycle helium cryostat, which operates at 2.5 K and is almost free of vibrations (a detailed description and a schematic drawing of the setup are provided in the Supporting Information). This low‐temperature environment allows us to conduct matrix isolation EPR experiments in hydrogen matrices in a more economic and sustainable fashion. As proof of principle, we studied the 2,2,6,6‐tetramethyl‐1‐piperidinyloxyl radical (**TEMPO**) in argon and *p*‐H_2_ matrices, respectively. The benefits of *p*‐H_2_ in EPR spectroscopy become even clearer by the characterization of a *P*‐centered mono‐radical showing nuclear‐spin interactions with several hydrogen nuclei, which we prepared by in situ photolysis of the corresponding phosphorus iodide.

**TEMPO** is a well‐known persistent radical within the EPR community, commonly used as a spin label, among other applications.^[^ ^24^, ^25^ ^]^ The unpaired electron is primarily located at the π* molecular orbital of the N–O bond, resulting in a triplet EPR spectrum caused by the hyperfine splitting with the ^14^N (*S *= 1) nucleus. The solution‐phase EPR spectrum of **TEMPO** exhibits three isotropic, symmetric bands, whereas in the rigid limit, such as in matrix isolation, the three bands become anisotropic. EPR spectroscopy is commonly used to study the polarity and proticity in the nitroxides’ environment by studying the *g_xx_* tensor of the nitroxide and the *A_zz_* tensor of the nitrogen nucleus, respectively.^[^ ^26^, ^27^ ^]^ Whereas the former requires multifrequency EPR spectroscopy for its accurate quantification, the latter can be estimated as half the separation between the outer lines of the X‐band spectrum (Figure 1).^[^ ^28^ ^]^ As an example, the nonpolar/aprotic limit was estimated at 33.6 G with di‐*t*‐butyl nitric oxide and 36.4 G under the high‐polar and high‐protic condition.^[^ ^29^ ^]^ Semiempirical calculations predicted both limits to be 33.6 and 37.0 G, respectively, for an NO spin‐labeled bacteriorhodopsin.^[^ ^27^ ^]^ Despite its large application in the condensed phase, the microsolvation of **TEMPO** has only been recently studied at low temperatures by Fourier‐transform infrared (FTIR) supersonic vacuum isolation jet spectroscopy.^[^ ^30^ ^]^

**Figure 1:**
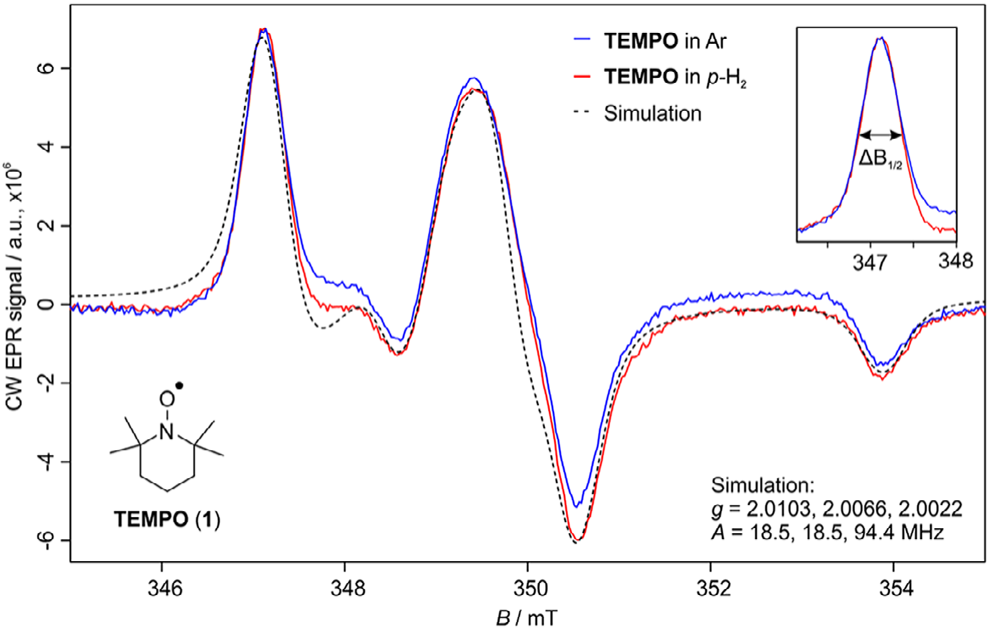
Comparison of the X‐band EPR spectrum of **TEMPO** (**1**) in argon (blue trace) and *p*‐H_2_ (red trace) matrices at 2.5 K. The spectra were recorded with attenuation of 59 dB, modulation amplitude (MA) of 0.5 G, and 100 scans. The spectrum of **TEMPO** was simulated with the parameters shown in the right‐bottom part of the figure. The inset shows a closer look at the low‐field line of **1** in both matrices.

We sublimed **TEMPO** from a storage vessel at −45 °C into our high‐vacuum chamber and mixed it with an excess of the matrix gas (argon or *p*‐H_2_). The mixture was subsequently deposited for 15 min onto a cold copper rod, directly attached to our cryostat (Picture S4) at 2.5 K. The presence of several matrix sites has been evaluated by comparing the linewidth of the sharper, isolated low‐field band. This effect was quantified as the full width at half maximum (FWHM) of the band centered at 347 mT of **TEMPO** (∆*B*_1/2_) and was estimated to be 13.35 ± 0.35 MHz in *p*‐H_2_ and 14.10 ± 0.35 MHz in argon (see Supporting Information for further details). These findings suggest a higher homogeneity in the *p*‐H_2_ matrix compared to argon. The spectrum of **TEMPO** in *p*‐H_2_ was simulated with the parameters shown in Table S1, simulation 1, showing a good agreement with the experimental spectrum (Figure 1, dashed trace; further details about the simulation are provided in the Supporting Information).

The analysis of **TEMPO** afforded some promising results regarding the properties of *p*‐H_2_ matrices. We recently reported the IR, UV/vis, and EPR spectroscopic characterization of the *P*‐dibenzophospholyl radical (**2**) as well as its nitrogen analog, the *N*‐carbazolyl radical, in solid Ar and partially (with IR and UV/vis spectroscopy only) in solid *p*‐H_2_.^[^ ^31^ ^]^ These radicals were generated from their iodo and nitroso precursors, respectively, showing clear differences regarding the photolysis conversion in Ar and *p*‐H_2_ matrices for iodo phosphine **3**. The generation of radical **2** in Ar matrices was obtained in low yield (10%) due to the matrix cage effect.^[^ ^31^ ^]^ The splitting patterns observed by EPR spectroscopy for the structurally similar *P*‐dibenzophospholyl radical (**2**) and the *N*‐carbazolyl radical differ in Ar. The spectrum is dominated by the splitting of the heteroatom into a doublet for **2** due to the nuclear spin of the P atom (*S* = ½) and a triplet for the carbazolyl radical due to the nuclear spin of the N atom (*S* = 1). These bands are further split into quintets for the carbazolyl radical in the experimental spectrum in agreement with theoretical predictions showing the larger hyperfine coupling constants (hfccs) with the pairs of hydrogen atoms in positions 1 and 3 (H_1_, H_1_', H_3_, and H_3_' in Table S3). The same is predicted for radical **2**, although the experimental spectrum in argon rather shows the splitting of the outer bands into triplets (Figure 2, blue spectrum).^[^ ^31^ ^]^ Therefore, we revisited the experiment and recorded the spectrum of **2** in different matrices with our new experimental setup.

**Figure 2:**
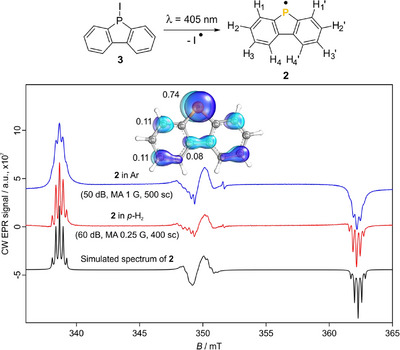
Comparison of the X‐band EPR spectra of radical **2** in argon (blue trace) and *p*‐H_2_ (red trace) at 2.5 K and its simulated spectrum with the parameters provided in Table S2 (black trace, further details in the Supporting Information). The generation of radical **2** from iododiphenyl phosphine **3** is depicted above the figure. The spin density of radical **2** at the UB3LYP‐D3/def2‐TZVP level of theory is shown together with the spin densities at the affected C and P atoms on the top part of the figure.

For a better comparison, the deposition conditions for precursor **3** (1 h at 90 °C) were kept constant for all experiments. Irradiation (405 nm, 5 min) of **3** isolated in *p*‐H_2_ at 2.5 K affords the spectrum of radical **2** (Figure 2, red spectrum) with a signal 10–12 times higher in intensity compared to Ar (vide infra, further details in the Supporting Information). Subsequent irradiation for 3 min does not induce any changes, suggesting complete precursor conversion after 5 min. This indicates that the low conversion (∼10%) observed in IR^[^ ^31^ ^]^ and EPR experiments in Ar is due to the matrix cage effect, which is absent in the softer *p*‐H_2_.^[^ ^13^ ^]^ The spectrum of **2** in *p*‐H_2_ exhibits a higher resolution, resulting in fully resolved quintets in the outer bands of the spectrum. The resolution of the bands in the central radical region is also higher in the *p*‐H_2_ experiment, as well as the more even baseline. This spectrum was simulated (Figure 2, black trace) using the parameters from Table S2, which yielded isotropic hfccs (*A*_iso_) for the protons in positions 1 and 3 of 7.8 and 7.2 MHz, respectively, in good agreement with the predicted values of 7.0 and 7.2 MHz.^[^ ^31^ ^]^ Therefore, the enhanced resolution of **2** in *p*‐H_2_ led to more accurate simulation parameters. The same set of parameters was used to simulate the spectrum in argon. Interestingly, the spectrum in argon was best fitted with a broadening of 0.15 mT compared to 0.055 mT required for *p*‐H_2_ (Figure S2). This nearly corresponds to a three‐fold increase in resolution in *p*‐H_2_ compared to argon. Annealing to 25 K of matrix‐isolated **2** in argon showed no resolution improvement, ruling out matrix inhomogeneity as the cause for the differences observed.

For a broader comparison, radical **2** was also generated in similar matrices to *p*‐H_2_ as *n*‐H_2_ and *normal*‐deuterium (*n*‐D_2_)^[^ ^32^ ^]^ to investigate the origin factors for the increased resolution. The comparison with *p*‐H_2_ could reveal if this relates to the matrix size and/or quantum solid nature of *p*‐H_2_. Radical **2** was also produced in neon to rule out argon‐specific matrix effects (Figure S3). Irradiation (405 nm, 5 min) of precursor **3** in *n*‐H_2_ matrix affords the spectrum of **2**, which increases by 15% after a further 10 min of irradiation. Therefore, the conversion is slower than in *p*‐H_2_, but much faster than in the other matrices tested (see Supporting Information for further details). The spectrum of **2** in *n*‐D_2_ shows both intensity and resolution ranging between Ar and *p*‐H_2_ (Figure S3). However, the spectrum of **2** in neon shows an even lower intensity than in argon, despite its smaller molecular size (Figure S3). The outer bands in Ne show sets of quintets, but the resolution in the central radical region is even lower than in Ar.

In summary, the photolysis yield is inversely related to the matrix host size except for neon, leading to the following series in decreasing order (relative intensities in parentheses, further details in the Supporting Information): *p*‐H_2_ = *n*‐H_2_ (1) > *n*‐D_2_ (0.41) > Ar (0.085) > Ne (0.006). The differences in sensitivity are related to the cage effect, affording lower photolytic yield in matrices like argon. Regarding the resolution of **2**, a similar trend was observed with a large improvement for both hydrogen matrices. These results not only show an increase in resolution in *p*‐H_2_ matrices compared to argon but also the possibility to obtain highly resolved spectra much faster than with our previous setup (Figure S4). No relevant differences were observed in the spectrum of radical **2** between *p*‐H_2_ and *n*‐H_2_ matrices. However, it should be noted that the spectrum of the *N‐*carbazolyl radical (**4**) looks the same in *p*‐H_2_ and argon matrices (Figure S5). We hypothesize that the larger hfccs of this radical (12 MHz, Table S3) can be better resolved in argon than those of radical **2** (∼7.5 MHz, Table S2). The resolution observed in *p*‐H_2_, related to the broadening of the simulation, remains similar for both radicals, whereas different parameters were required to fit the spectra of each radical in argon (Figures S2 and S6). Therefore, the increased resolution in *p*‐H_2_ matrices may be related to the magnitude of the hfcc of the studied radical.

In this communication, we report the first matrix‐isolated EPR spectra of larger radicals (in the context of matrix isolation studies) in *p*‐H_2_ matrices. So far, the few EPR experiments performed in *p*‐H_2_ matrices required the use of expensive liquid helium in a non‐sustainable fashion and were focused on small molecules such as the methyl radical. Our upgraded matrix setup allows recording EPR spectra on a daily basis at a low operating cost using a powerful closed‐cycle helium cryostat. This EPR matrix setup allows the investigation of larger molecules in *p*‐H_2_ matrices, which can now be compared to IR and UV/Vis matrix studies under the same experimental conditions.

Experiments performed with the **TEMPO** radical show diminished matrix effects in *p*‐H_2_ matrix compared to argon. Moreover, we reported the first EPR experimental and simulated spectra of matrix‐isolated **TEMPO**. The characterization of a *P*‐centered π radical shows higher resolution in hydrogen matrices compared to argon, neon, and deuterium. This enhancement is approximately three times greater in *p*‐H_2_ than in an argon matrix, enabling the more accurate determination of hfccs, which are crucial for molecular characterization and benchmarking gas‐phase calculations. In the field of astrochemistry, the acquisition of accurate hfccs is fundamental for the interpretation of rotational spectra.

The data provided in this work suggest that the enhancement in sensitivity relates to the cage effect and the improved resolution originates from the softness and size of the matrix host. Further investigations with other paramagnetic species remain crucial to clarify these questions.

## Supporting Information

The authors have cited additional references within the Supporting Information.^[^ ^33^, ^34^, ^35^, ^36^, ^37^, ^38^, ^39^, ^40^ ^]^

The Supporting Information File to this Article Includes:

Considerations for the design of a matrix EPR setup, description of the upgraded matrix EPR setup, simulation of the **TEMPO** radical in *p*‐H_2_ matrix, quantitative comparison of the resolution of radical **2**, EPR spectra of **2** in different matrices, EPR spectra of **4** in argon and *p*‐H_2_ matrices, method of *o*/*p*‐H_2_ determination, methods, and optimized geometries (PDF).

## Conflict of Interests

The authors declare no conflict of interest.

## Supporting information



Supporting Information

## Data Availability

The data underlying this study are available in the published article, in its Supporting Information, and openly available in RESOLVdata at https://doi.org/10.17877/RESOLV‐2025‐MCX9DTIH.
