# Supplementary material for: Enhanced Resolution in EPR Spectroscopy Using para‐Hydrogen Matrices
Source: Angew Chem Int Ed Engl. 2025 Nov 19;65(6):e18517. doi: 10.1002/anie.202518517 (PMC12865239; doi:10.1002/anie.202518517)
Supplement: Supplementary file 1 — Supporting Information [file ANIE-65-e18517-s001.pdf]

Supporting Information

Enhanced Resolution in EPR Spectroscopy Using *para*-Hydrogen Matrices

Adrián Portela-González, Wolfram Sander and André K. Eckhardt*

Lehrstuhl für Organische Chemie II, Ruhr-Universität Bochum, 44801 Bochum (Germany); Andre.Eckhardt@ruhr-uni-bochum.de

Table of Contents

Considerations for the design of a matrix EPR setup S2
Description of the upgraded matrix EPR setup S3
Simulation of the **TEMPO** radical in *p*-H_2 matrix S13
Quantitative comparison of the resolution of radical **2** S15
EPR spectra of **2** in different matrices S16
EPR spectra of **4** in argon and *p*-H_2 matrices S20
Method of *o*/*p*-H_2 determination S23
Methods S25
Optimized geometries S26
References S27

Considerations for the design of a matrix EPR setup

Before describing the upgraded setup, it is important to mention the observed difficulties when designing and operating a matrix EPR setup compared to other matrix (IR and UV/vis) setups. First, the sample should be inserted into a resonator with a small opening cavity. For example, we use a standard X-band ER4102ST resonator with vertical openings of 11 mm into the cavity. Therefore, the sample must be kept separated from the deposition unit contrasting with IR setups where the head is kept in a cubicle of approximately with 20 cm sides. Therefore, different parts of the matrix head must be independently mobile to allow for deposition and measurement at a different position. It should also be taken into account that experiments are performed between two magnets and any magnetic part close to the central position may either disturb the magnetic field during the measurement or, in worst case, feel attracted to the magnet causing potentially movement of the head with the derived dangers of possible collisions with the magnet/resonator leading to breakage. Additionally, the EPR experiment requires the critical coupling of the cavity for accurate measurements. That makes this technique extremely sensitive to any changes during the measurement such as microvibrations of the inner part of the head caused by the cryostat. We had experienced this issue with our previous setup, and this led sometimes to spikes in the spectrum matching the cryostat frequency or even to decoupling of the resonator completely stopping the measurement. The sensitivity of the spectrometer to these changes increases whenever measuring at higher microwave powers.

Alternatively, the use of liquid helium is not only expensive but other problematic scenarios should also be considered. For example, a simple setup such as a Dewar filled with the cold liquid (either helium or nitrogen) may lead to slow evaporation, thus generating bubbles within the liquid that move the sample tube leading to the same issue we have observed with the head vibrations, thus not allowing to record spectra. This can be circumvented with the use of a helium cryostat. However, other problems may also arise. We have experience with such a setup (the cold liquid is evaporated from a Dewar and flown into a finger Dewar with the sample) used with liquid nitrogen and the lowest temperature we can achieve is 83 K, slightly higher than that of liquid nitrogen (77 K). A similar problem is likely to occur with liquid helium, thus increasing the temperature above that of liquid helium (4 K) also requiring extra equipment to maintain the setup within the temperature required to work with hydrogen matrices (<4.5 or 5.0 K).

Description of the upgraded matrix EPR setup

The setup consists of a two-stage RK-415D2 cold head (1.5 W) from Sumitomo Heavy Industries supported by a F-50 Sumitomo cryostat. This setup is based on previously described matrix setups described in detail by Dunkin, where also useful information about the matrix isolation technique is provided.[1] The matrix head is attached to a metal column (Picture S1) that allows vertical movement to either enter the resonator cavity or to set it at different heights to perform other action such as irradiation. A heating block made out of oxygen-free copper is screwed to the bottom of the cold head (Picture S2) with a piece of indium (100 µm thickness) connecting both surfaces to ensure good thermal conductivity. The heating block contains a resistance to heat the matrix during matrix experiments as well as a non-magnetic temperature sensor to read the temperature without interfering with the magnetic field during the experiments. The copper rod (Picture S3, also made from oxygen-free copper) is screwed into the heating block with a piece of indium between them. An aluminum irradiation shield is screwed to the top of the cold head, which covers all the components below except for the bottom part of the copper rod required for measuring (Picture S4). Care should be taken for the design of the shield with the space limitations of the EPR matrix setup. The shield is crucial to achieve the required low temperatures. In its absence, our setup could not go below 6 K.

The whole setup is covered by a brass shroud (head cover) consisting of two threaded parts allowing to vary the length of the outer structure so that the copper rod can be aligned with the deposition unit for deposition and then placed into the quartz tube for measurements or irradiation (Picture S9). This shroud is connected to an intermediate piece that has a thinner bottom part so that it can be placed into the magnets for measurements (Picture S5). This bottom part has a cubic structure at the bottom to which the deposition unit (also pyrolysis oven, etc.) can be attached. The bottom of the cubic part has a circular opening to which a quartz tube glued into a brass holder is added to perform the measurements (Pictures S6-7). The quartz tube has a diameter of 9.2-9.3 mm so it tightly fits within the 11 mm cavity of our ER 4102ST X-band resonator. Rotating the shroud (using the handles in the top part, allowing ¼ of a turn before repositioning to the next handle) requires the bottom part to be fixed to the supporting column so that both parts of the shroud do not rotate together (Picture S8). The head is pumped by a scroll pump and a Turbopump reaching a pressure of $3 \cdot 10^{-5}$ mbar at room temperature and $3 \cdot 10^{-6}$ mbar at 2.5 K.

Most of the parts are built in brass to avoid interaction with the magnetic field. The inside parts are made from oxygen-free copper since it has better thermal conductivity than brass and also

does not interfere with the magnetic field. Both brass and steel deposition units have been used indistinctively for the experiments. Additional information about the parts is provided in the picture descriptions.

Additional improvements compared to the previous setup are not only related to the lowest temperature but also to the decrease of vibrations of the matrix head. The upgraded setup allows measuring spectra at the highest microwave power (203.6 mW, 0 dB) allowed by our setup, whereas measurements below 6.4 mW (15 dB) were not possible with the previous setup. This difference would allow to increase the sensitivity of our setup given that saturation does not occur at those high microwave powers (depending on the system under study). Such example has not been studied within this manuscript, but it is an additional advantage offered by the upgraded setup.

The different parts of the matrix head and the whole setup (including magnets and supporting column) with their dimensions can be accessed in the PDF file provided with the supporting files.

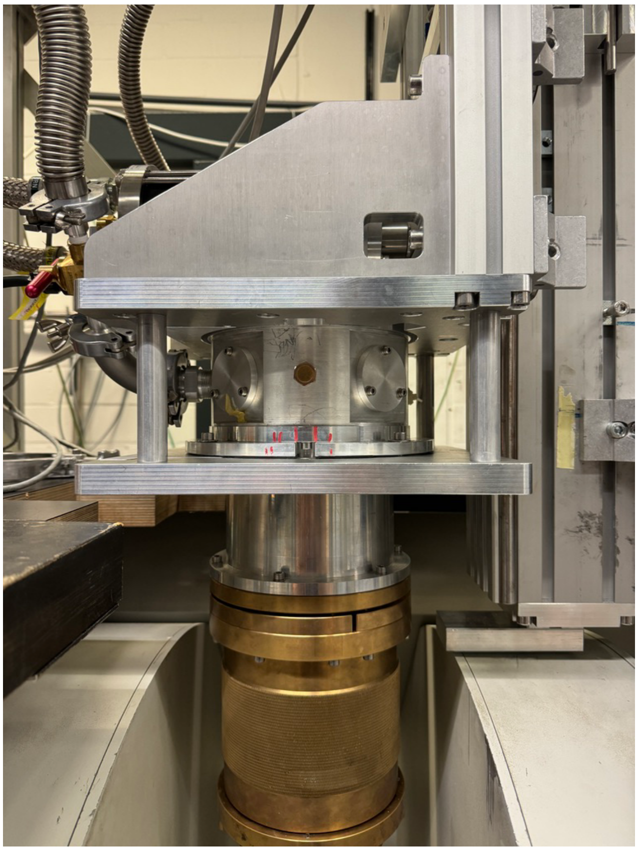

Picture S1. View of the matrix head connected to the supporting column (on the right). In this picture, the brass shroud is at his lowest length prepared for measurement.

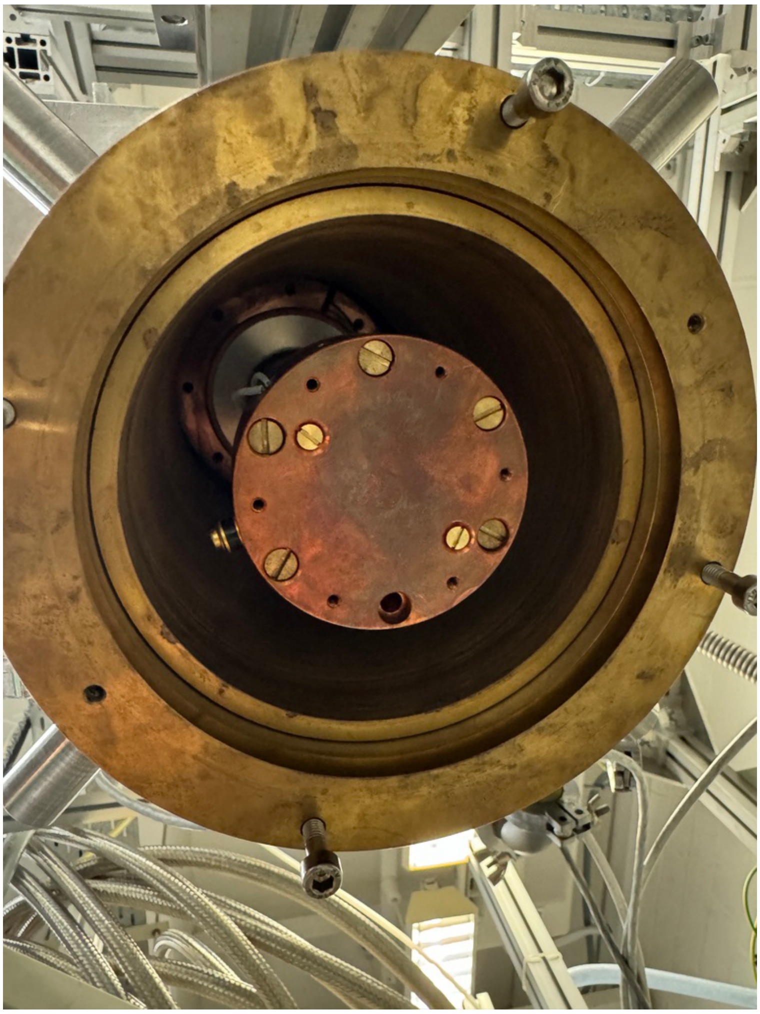

Picture S2. Bottom view of the inside of the top shroud piece. In the inside, the copper heating block can be seen.

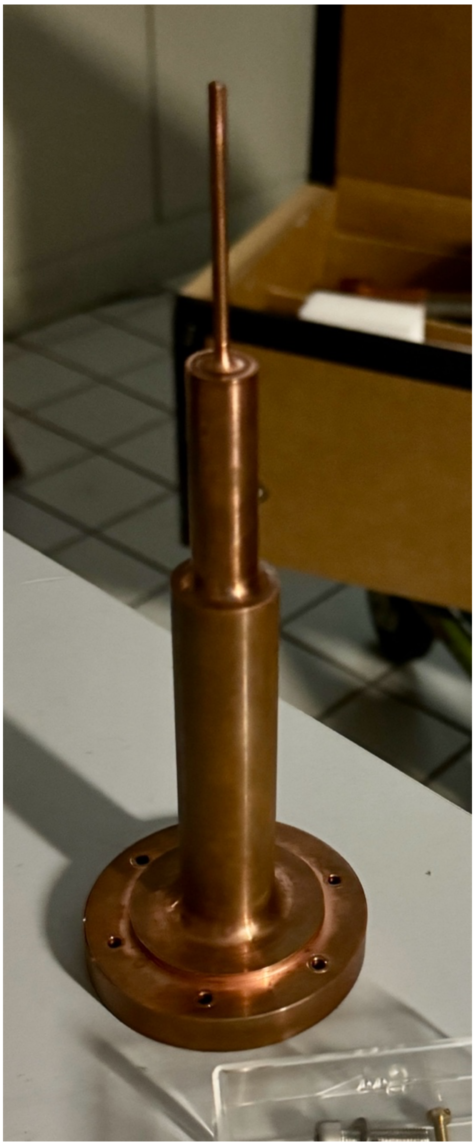

Picture S3. Copper rod. This piece consists of a single piece of oxygen-free copper with different diameters depending on the height to not interfere with the different parts of the shroud, which is getting thinner towards the bottom while maintaining good thermal conductivity.

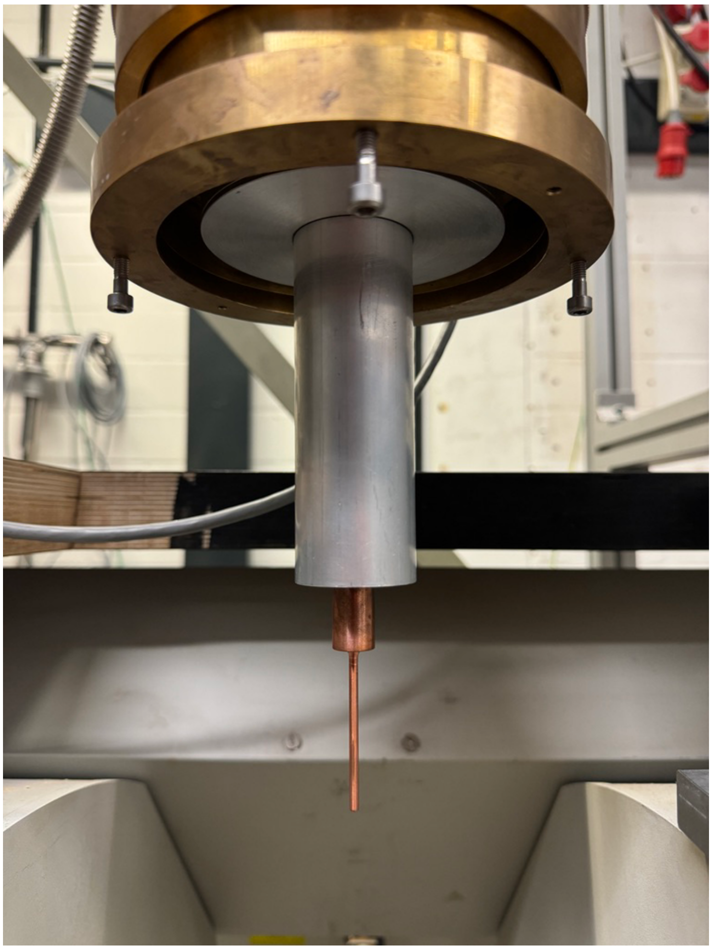

Picture S4. View of the aluminum shield covering the inner structure except the tip of the copper rod.

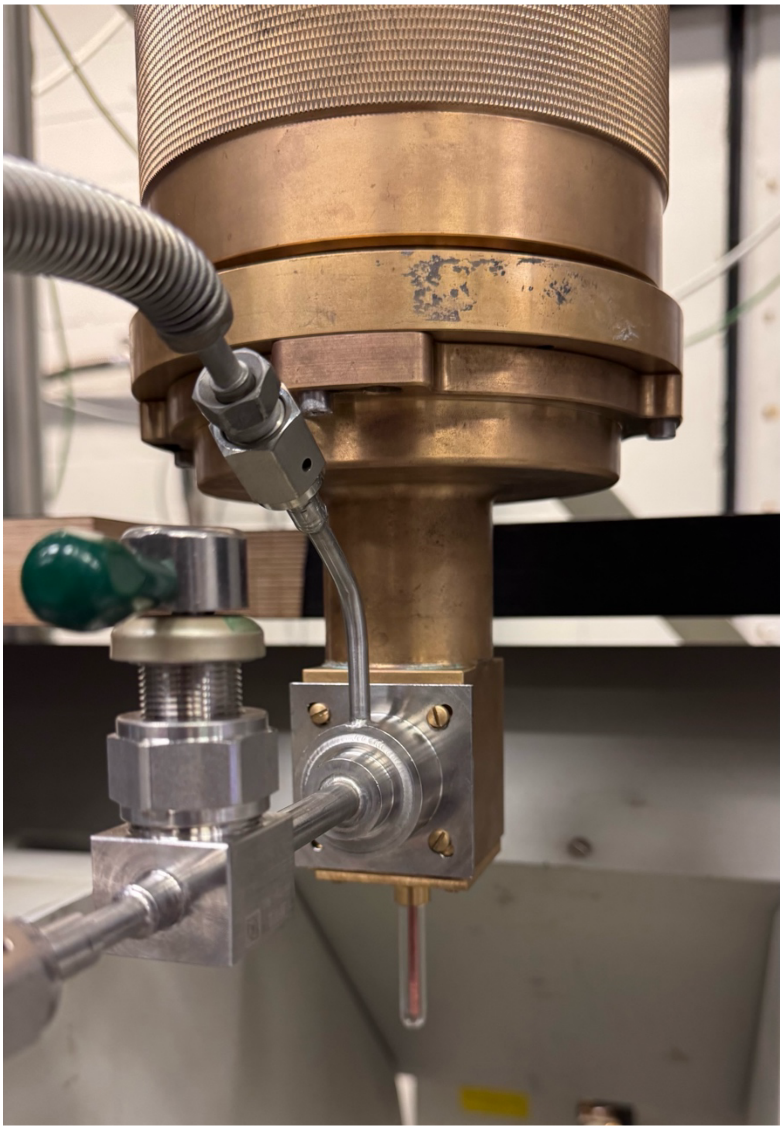

Picture S5. Bottom part of the matrix head showing the lower piece of the shroud and the holder with the quartz tube, where the copper rod can be seen in its measurement position. For deposition, the length of the shroud is increased so the copper rod is aligned with the deposition unit.

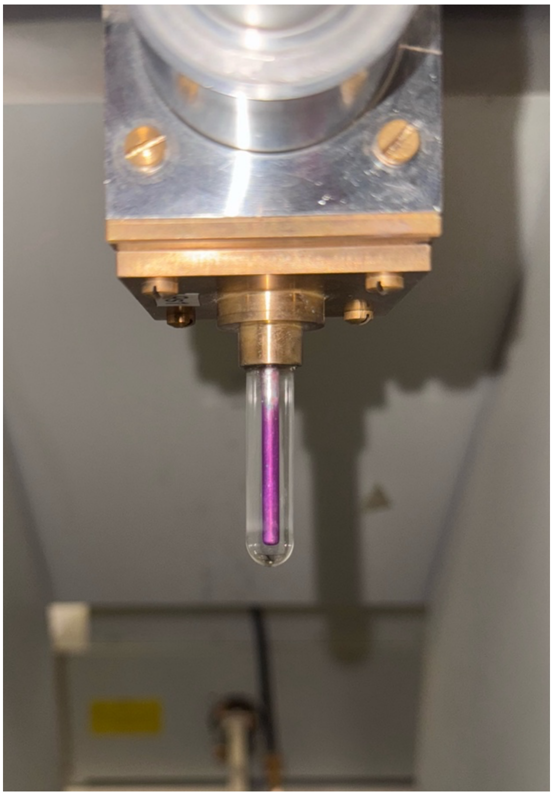

Picture S6. View of the copper rod inside the quartz tube containing the *N*-carbazolyl radical in Ar, which shows a purple coloration.

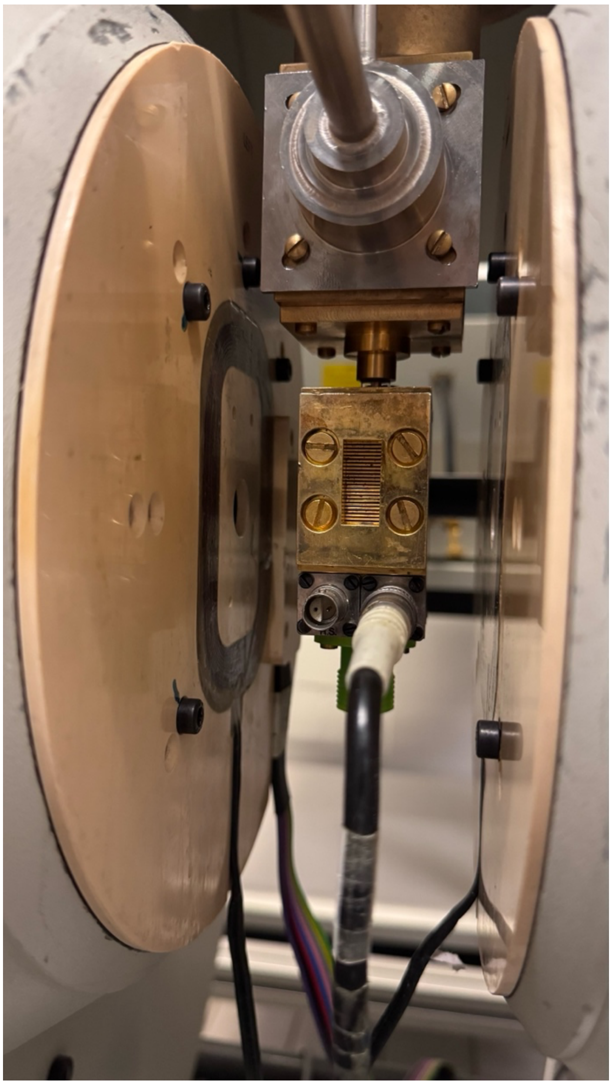

Picture S7. View of the matrix head inserted into the resonator cavity for measurement.

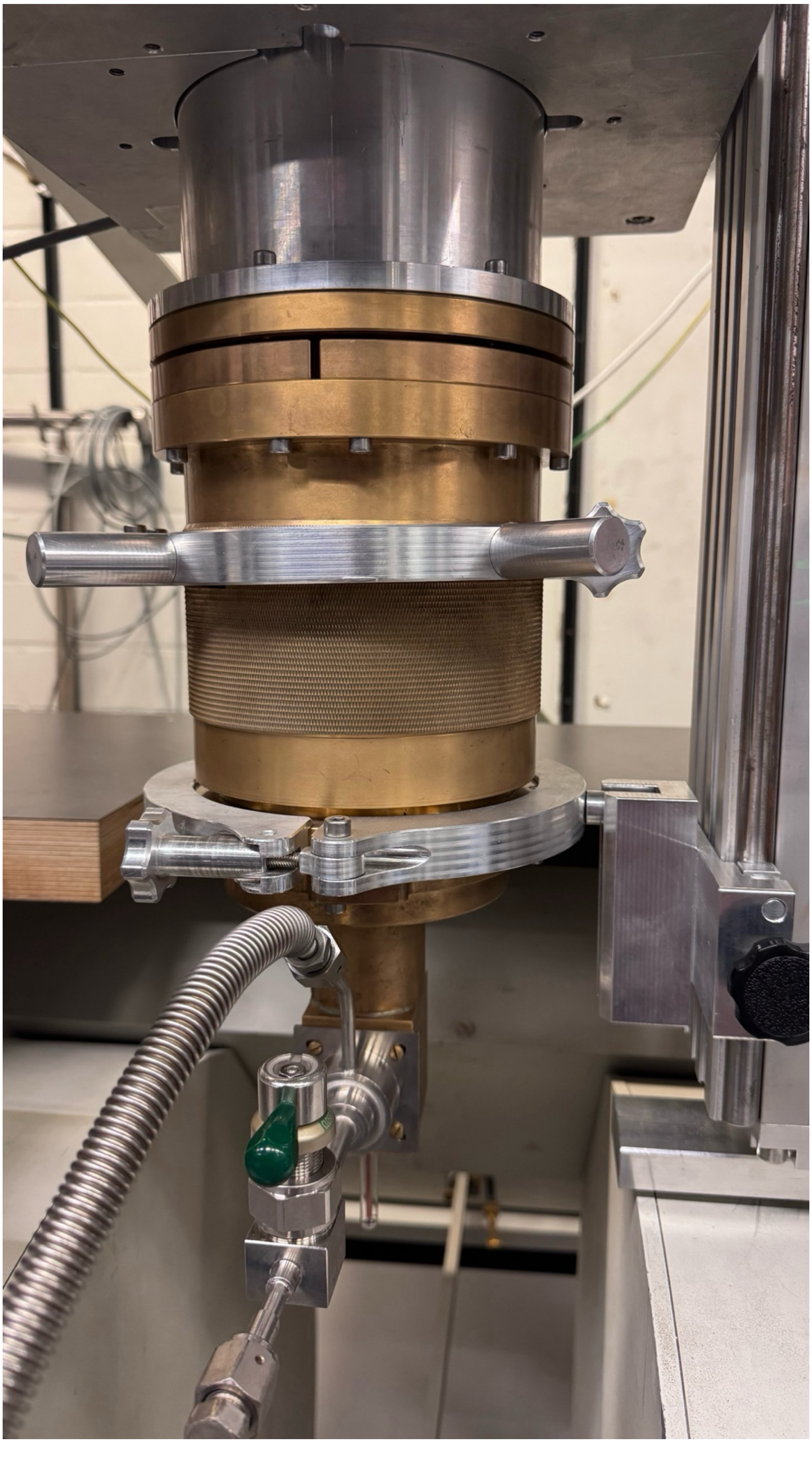

Picture S8. Mechanism required for rotating the shroud. The bottom part is fixed to the supporting column, and the top handles can be used to shift the bottom part of the shroud lower so that the copper rod is aligned with the deposition unit.

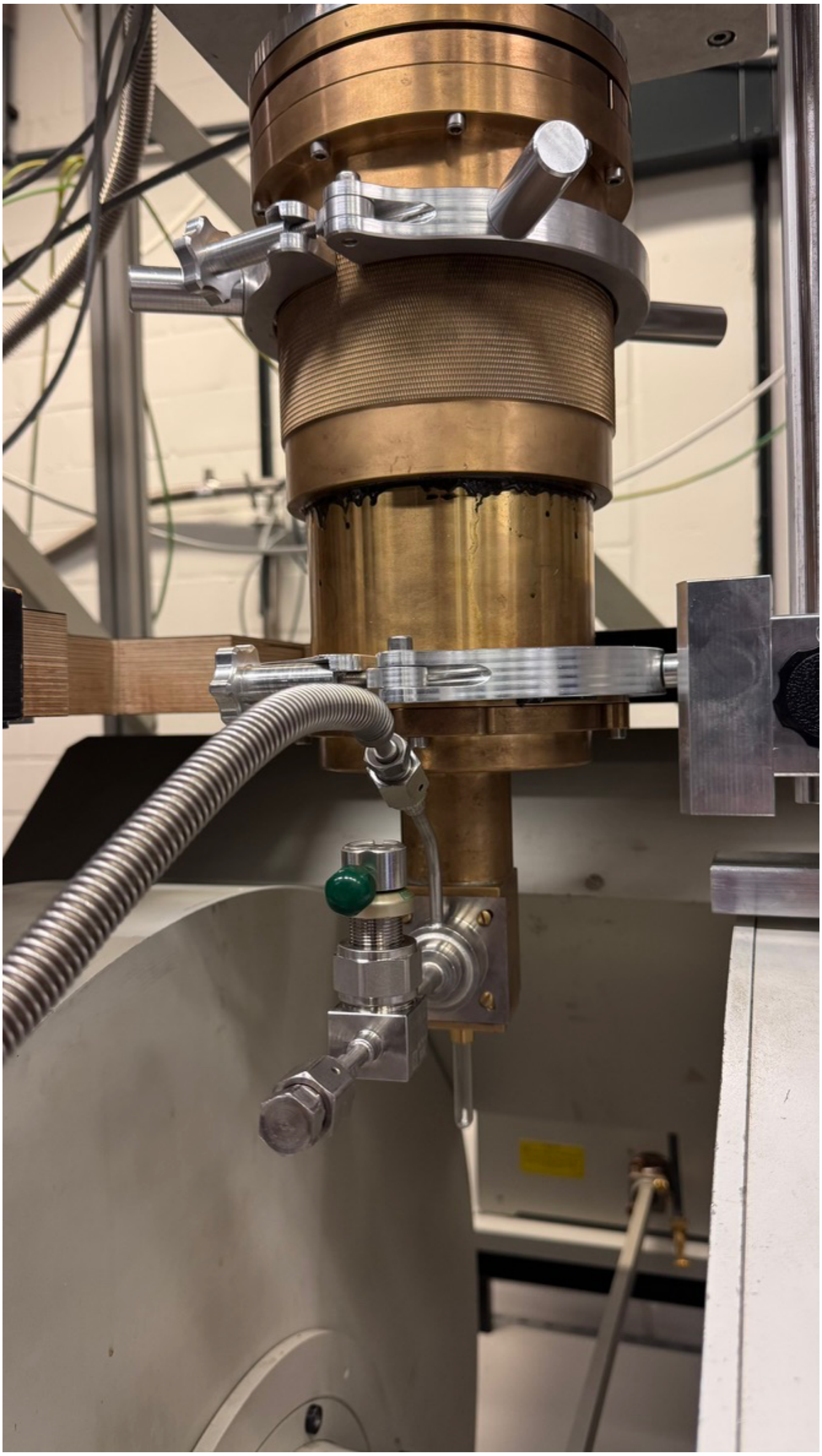

Picture S9. View of the extended shroud required to place the copper rod aligned with the deposition unit for sample deposition.

Simulation of TEMPO radical in *p*-H_2 matrix

The simulation of the spectrum of TEMPO was first attempted using parameters similar to those available in the literature. The A_{zz} tensor of the hyperfine coupling with the N atom was directly extracted from the spectrum (94.4 MHz) by measuring the distance from the absolute maxima between the high-field and low-field bands as reported in the literature.[2] This value corresponds to 33.7 G, close to the non-polar limit mentioned in the literature (33.6 G). The best fit of the simulation of **TEMPO** was achieved with the parameters shown in Table S1, Simulation 1 (also shown in Figure 1, dashed line). However, noticeable differences have been observed with the spectra reported for different nitroxides in frozen media. These differences will be addressed and discussed in the following. The experimental spectra of a variety nitroxides has been reproduced using often equal (or almost) values for the A_{xx} and A_{yy} tensors, ranging between 0.4 and 0.6 mT (11.2 to 16.4 MHz).[2] However, higher hyperfine coupling constants in these axes have been detected for example for OH-TEMPO, for which Savitsky *et al.* reported A_{xx} = 19.45 MHz and A_{yy} = 18.90 MHz.[3] These values approach more those used in this study to reproduce the spectrum of **1** (Figure 1 and S1 and Table S1, Simulation 1). The centering of the high- and low-field peaks is good in all the spectra considering the obtained A_{zz} value from the spectrum. However, noticeable differences can be observed regarding the intensity ratio of the positive signals labelled as **a** and **c** and the relative position of the smaller signal labelled as **b** with respect to **a** and **c**.

In this case, we assume A_{xx} = A_{yy} and the best results were obtained in the region of 18.5/19.0 MHz (Sim. 1-2). These values ensure that the small signal labelled as **b** is sitting at the right position with its maximum in between **a** and **c** slightly shifted towards the signal **a**. However, using lower values closer to those often used in the literature (Sim. 3) leads to a disagreement with signal **b**.

Additionally, the relative intensities of the signals **a** and **c** is slightly influenced by the A_{xx} and A_{yy} tensors and is strongly influenced by the ratio of g_{xx} tensor compared to g_{yy} and g_{zz}. The higher relative intensity of the signal **a** with respect to **c** (as observed in the experimental spectrum) could only be achieved at g_{xx} values higher than 2.01. It should be noted that in the literature, nitroxides in apolar aprotic media usually show g_{xx} values in the order of 2.008 to 2.009 in most cases.[4] Therefore, values higher than 2.009 are unusually large, mainly considering that g_{xx} shifts usually ± 0.001 from the polarity changes between apolar solvents like toluene and water.[4,5] However, the simulation of the spectra is best achieved with such high values > 2.01 for g_{xx}. Changes of g_{yy} and g_{zz} were also attempted but the best simulation

was achieved with the parameters used for simulation 1. Nevertheless, it should be mentioned that the obtention of accurate g_{xx} values requires the use of high-frequency EPR spectra and X-band does not fulfill that condition. In any case, we wanted to point out that the values reported in this manuscript appear to be strangely large. It is unclear whether this is due to the use of gas matrices, or the simulation is not accurate due to the low frequency used for the experiment.

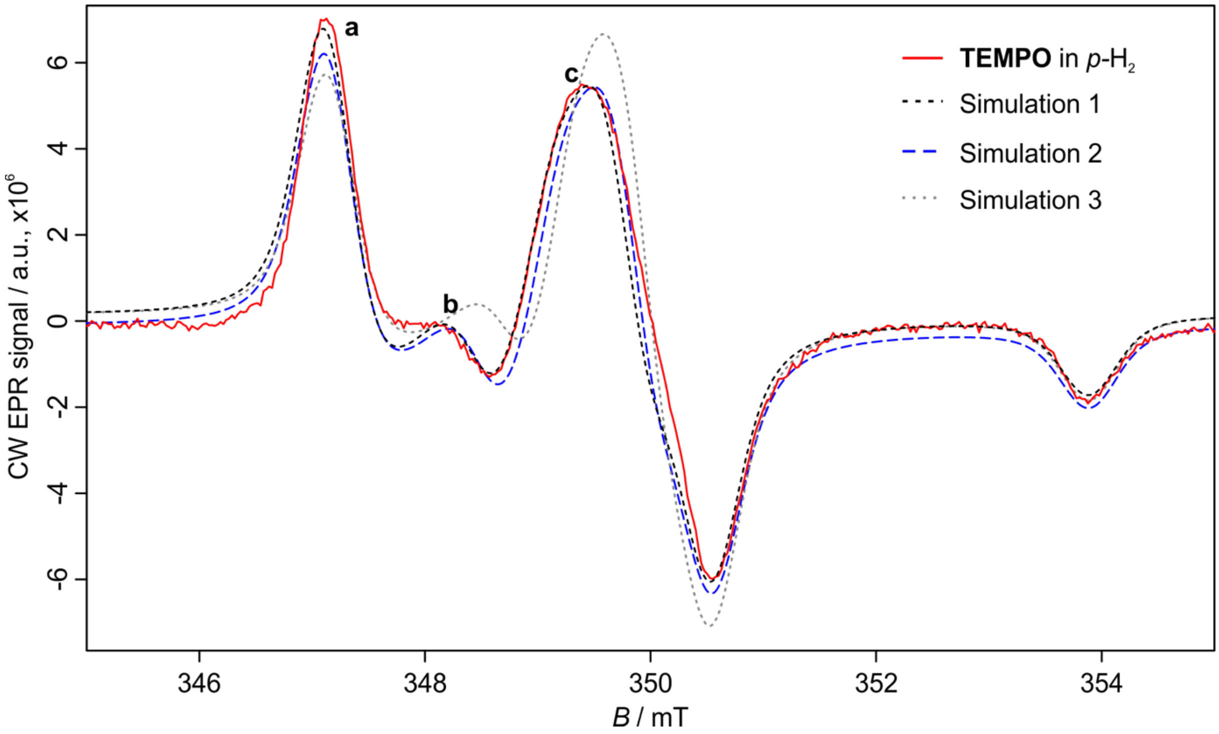

Figure S1. Matrix-isolated X-band EPR spectra of **TEMPO** (**1**) in *p*-H_2 at 2.5 K and their simulated spectra (dashed traces) using different parameters (see Table S1). The broadening parameters were kept constant using Voigt broadening, which combines a Gaussian broadening of 0.35 mT and Lorentzian broadening of 0.20 mT. Simulation 1 corresponds to the simulated spectrum shown in Fig. 1. The different peaks discussed in the text are labelled as **a**, **b**, and **c**.

Table S1. Parameters utilized for the simulated spectra shown in Figure S1. The hyperfine coupling constants (*A*) are shown in MHz.

Simulation	g_{XX}	g_{YY}	g_{ZZ}	A_{XX}	A_{YY}	A_{ZZ}
**1**	2.0103	2.0066	2.0022	18.5	18.5	94.4
**2**	2.0099	2.0062	2.0022	19.0	19.0	94.4
**3**	2.0090	2.0057	2.0022	16.4	16.4	94.4

Quantitative comparison of the resolution of radical 2

The simulated spectrum of **2** in *p*-H_2 (Figure 2, black trace) was simulated with the parameters included in Table S2. The fitting is better in the more intense low-field quintet than in the high-field quintet, where a slight shift of 0.15 mT can be observed for all the bands. Additionally, the signal in the center of the radical region could not be easily fitted due to the large number of parameters to optimize.

Table S2. Parameters utilized to simulate the spectrum of *P*-dibenzophospholyl radical **2** in *p*-H_2 matrix**.** The hyperfine coupling constants (*A* or hfcc) are given in MHz and the broadening used was 0.055 mT. The hfcc of the protons are provided as their full 3x3 matrices with their signs kept from the calculations. The corresponding prime protons have the same parameters with their sign inverted in the out-of-diagonal terms. The sign of the hfccs has not been experimentally determined and has been kept as obtained from the ORCA calculation.

P-dibenzophospholyl radical **2**

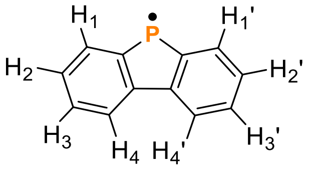

Tensor	x	y	z	Isotropic
g	2.0023	2.0035	2.0080	2.0046
A(P)	–21	–24	662	206
A(H_1)	$\begin{pmatrix} -8.0 & 0 & 0 \\ 0 & -8.5 & -3.5 \\ 0 & -3.5 & -7.0 \end{pmatrix}$			-7.8
A(H_2)	$\begin{pmatrix} 0.1 & 0 & 0 \\ 0 & 2.0 & 0 \\ 0 & 0 & 2.5 \end{pmatrix}$			1.5
A(H_3)	$\begin{pmatrix} -8.0 & 0 & 0 \\ 0 & -7.0 & 2.5 \\ 0 & 2.5 & -6.5 \end{pmatrix}$			-7.2
A(H_4)	$\begin{pmatrix} 0.1 & 0 & 0 \\ 0 & 2.2 & -0.1 \\ 0 & -0.1 & 1.8 \end{pmatrix}$			1.4

This set of parameters were used to simulate both the experimental spectra in argon and in *p*-H_2 matrices to quantify the improvement in resolution observed between both matrices. This analysis was performed focused on the low-field intense quintet, which has been very well reproduced by the simulation and is a good measure of the spectral broadening. The fitting was performed assuming a Lorentzian broadening to ease the comparison between both spectra with a single parameter, which afforded satisfactory results for both matrices. The broadening was optimized both takin into account the linewidth of the recognizable signals and the fitting of the

area between two lines. The optimal broadening values obtained were 0.150 mT for argon and 0.055 mT for p-H_2. This suggests that the improvement in resolution afforded by p-H_2 is roughly a factor of 3 higher than that obtained in argon.

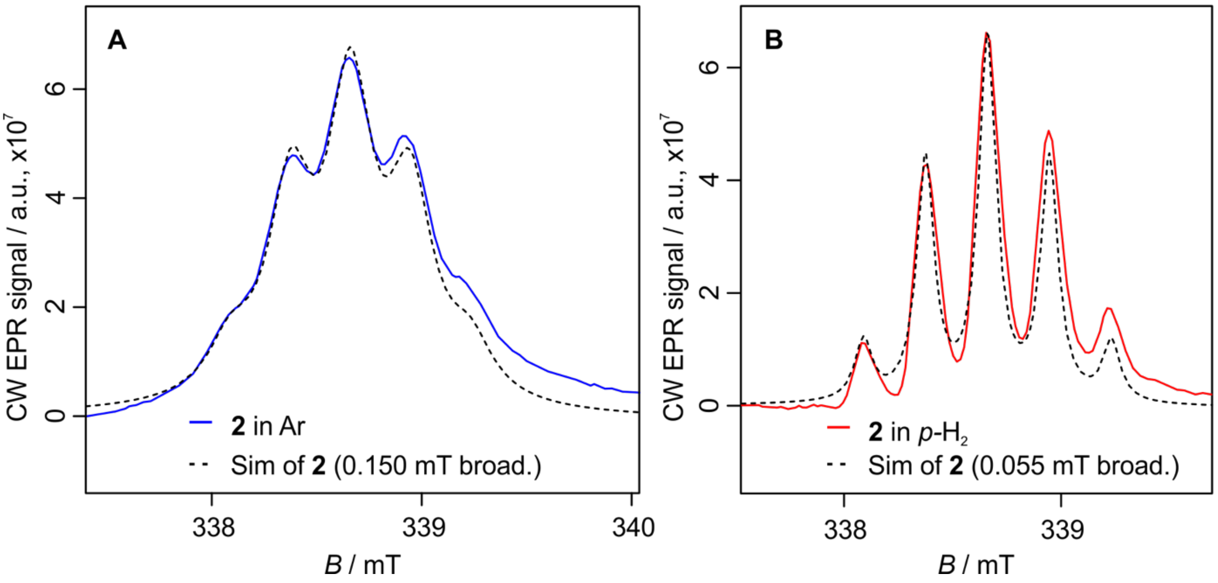

Figure S2. Experimental and simulated spectrum of radical **2** focused on the low-field wing signal. **A.** Experimental spectrum of **2** in argon matrix (blue trace) and simulated spectrum obtained with the parameters shown in Table S2 using Lorentzian broadening of 0.15 mT (dashed trace). **B.** Experimental spectrum of **2** in p-H_2 matrix (red trace) and simulated spectrum obtained with the parameters shown in Table S2 using Lorentzian broadening of 0.055 mT (dashed trace, the same as used for Figure 2, dashed trace).

EPR spectra of 2 in different matrices

The EPR spectrum of *P*-dibenzophospholyl radical (**2**) in different matrices was measured in different matrices after deposition under the same conditions (1 h deposition at 90 °C). Irradiation of **3** in p-H_2 and n-H_2 reached its maximum intensity after 5 min and 15 min irradiation, respectively. For the other matrices, the intensity was lower than in hydrogen matrices. Therefore, we can consider that complete conversion of the precursor was not achieved. However, the irradiation time was kept constant to avoid the formation of radical impurities, which were observed in Ar matrices after long irradiation times (up to 3 days). The irradiation time was kept to 15 min for deuterium and argon matrices. In the case of argon, a higher power light emitting diode (LED) was used. After 15 min irradiation (405 nm, low power) of **3** in neon, the conversion was so low that subsequent irradiation with the higher power LED for 15 min was performed. Nevertheless, the intensity of radical **2** in neon is clearly lower than that obtained in argon matrix.

The spectra in hydrogen could be recorded at the lowest microwave power achievable with our setup (60 dB, 0.0002 mW) for optimal resolution, whereas the other spectra were showing very

low intensity and the appearance of artifacts and had to be recorded at higher microwave powers (see the caption of Figure S3). The same occurs with the modulation amplitude (MA) that was kept at 0.25 G for the hydrogen spectra but was increased for the other matrices to either 0.5 G (in *n*-D_2) or 1 G (in Ar and Ne). The number of scans was adapted to the conditions of each experiment to obtain the best signal-to-noise ratio possible.

Since all spectra were recorded in the linear regime, they are quantitatively comparable. Given that the same conditions were used for deposition in all experiments, the amount of precursor present in each matrix should be the same in all experiments. The different parameters used for each experiment (microwave power, modulation amplitude and number of scans) were then normalized with respect to *p*-H_2 to have visually comparable data of the signal intensity and resolution obtained in each matrix. Considering the difference in signal intensity, the spectra in the matrices different than hydrogen were scaled up (scaling factor included next to the matrix material) to show the spectra in a comparable manner. Therefore, the signal-to-noise ratio (SNR) is not comparable between the difference matrices. The SNR is slightly affected by small changes in the parameters used for each experiment and more strongly affected by the difference in signal intensity observed in the different matrices. The latter is related to the vulnerability of the matrix material to the cage effect. Thus, those matrices with lower signal intensity required higher scaling factors and lower SNR compared to the other spectra.

The direct comparison of the different matrices is not straightforward because the spectra with lower resolution have higher integration areas than that higher resolution. An estimation of the intensity observed for the different matrices was performed relative to *p*-H_2. by overlapping both spectra after applying the correction for the difference in intensity related to the different parameters optimized for each matrix and adjusting the maxima of the signals that can be distinguished. There are small differences if the intensity is compared in the low-field signals or those in the central radical region. The observed intensity in *n*-D_2 corresponds to 37% on the left flank signal and 45% of the central radical signal. For Ar, the same values correspond to 7.2% and 9.5% respectively, whereas for neon both lines lie below 1% of the expected intensity (0.5% and 0.6%, respectively). Averaged values from those mentioned here averaging the outer and center signals yields have been included in the main manuscript to quantify the differences in signal intensity observed in each matrix.

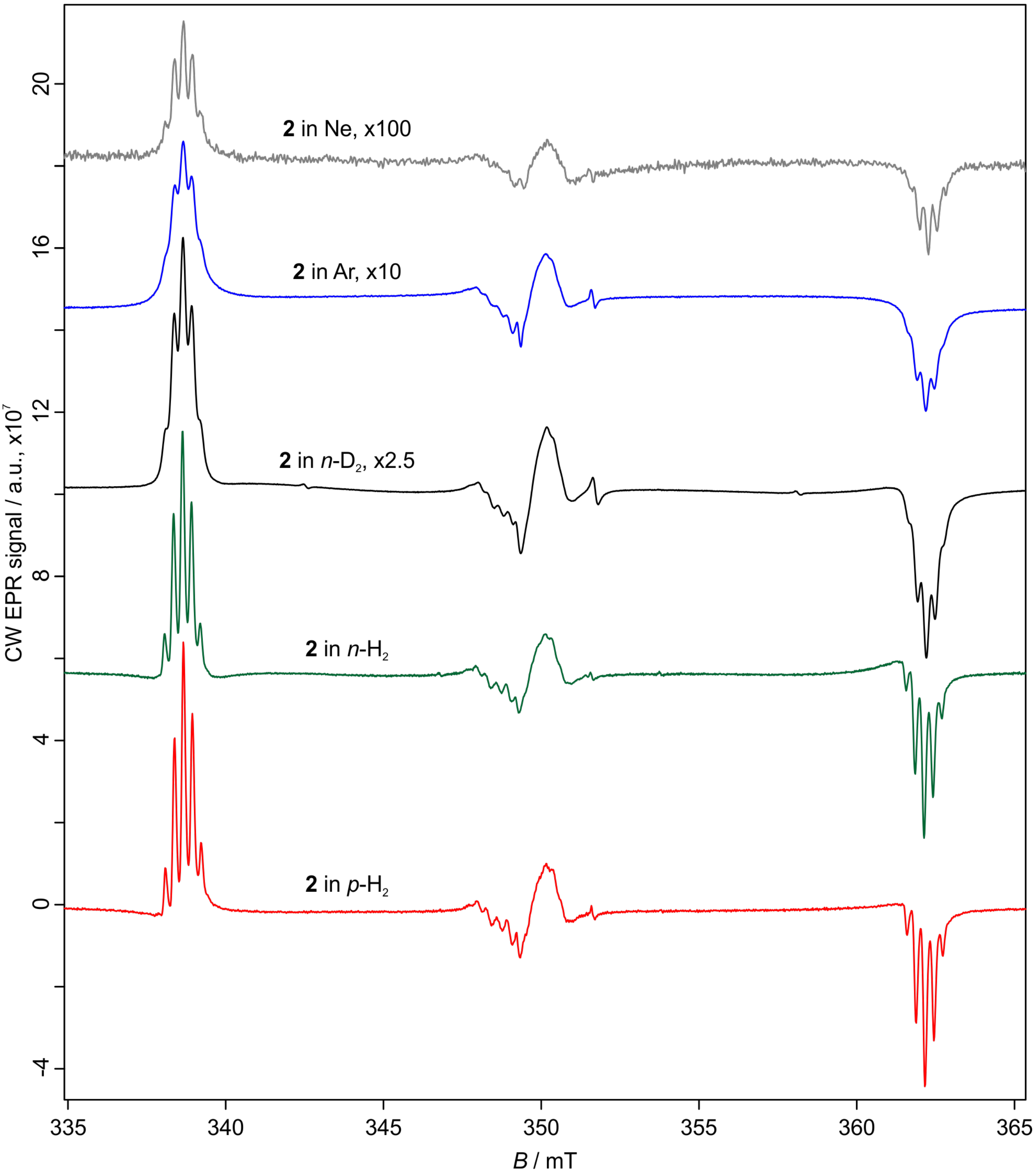

Figure S3. Matrix-isolated X-band EPR spectra of *P*-dibenzophospholyl radical **2** in different matrices at 2.5 K. The parameters used for recording each spectrum were the following for each matrix: *p*-H_2 (attenuation 60 dB; MA 0.25 G, 400 scans), *n*-H_2 (60 dB, 0.25 G, 300 scans), *n*-D_2 (50 dB, 0.5 G, 400 scans), Ar (50 dB, 1.0 G, 500 scans), and Ne (45 dB, 1.0 G, 300 scans). The spectra are normalized with respect to *p*-H_2 for the different parameters used. Then, the spectra with lower intensity are scaled up for a better resolution comparison between the different matrices applying a correcting factor mentioned next to the matrix material.

Quick measurements of radical **2** in *p*-H_2 also show a great improvement in both intensity and resolution compared to argon. A spectrum of **2** recorded after 5 min irradiation of precursor **3** of 5 scans (requiring solely 5 min) shows already a better resolution than the overnight scan recorded in Ar (13 h) after optimization of the conditions and parameters (Figure S4). The

signal-to-noise ratio is higher in the case of p-H_2 due to the lower number of scans recorded (5 vs. 500). Nevertheless, the resolution is higher both in the outer signals and in the radical region for the quick spectrum recorded in p-H_2. These results show that not only there is an increase on the resolution of radical **2** in p-H_2 but also high-quality spectra can be obtained within very few minutes (5 min irradiation and 5 min scan) compared to argon (13 h scan).

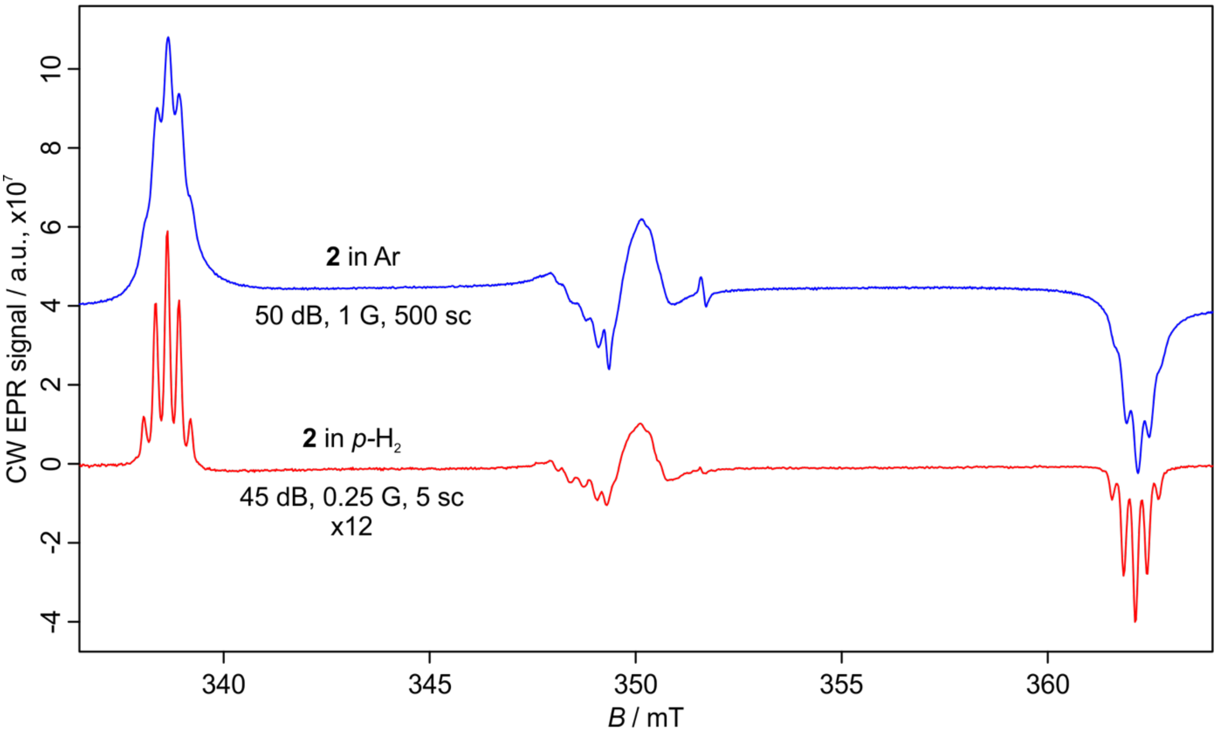

Figure S4. Matrix-isolated X-band EPR spectra of radical **2** in argon (blue trace) and p-H_2 (red trace) matrices at 2.5 K recorded with different parameters (shown in parenthesis within the figure). The spectrum in p-H_2 has been increased by a factor of 12 so the intensities of both spectra are comparable.

The expected ratio for the different parameters should show a ratio of 18.5:1 higher for the spectrum in Ar, however, this is not the case. Instead, the signal of **2** in Ar based on the lower-field signal corresponds to 5.9% of that of p-H_2. This value is lower than that of 7.2% obtained after the long scan. If we consider this difference not negligible and taking into account that all the spectra were checked for saturation, it could be argued that there is a difference in intensity of the spectrum of **2** in p-H_2 over time. We can think of two arguments leading to this behavior: a) slow diffusion of molecules leading to quenching of radical **2** e.g. by dimerization, or b) slow evaporation of the outer layer of the matrix over long periods of time. For the sake of reproducibility of the experiments in different matrices, the deposition performed with p-H_2 was much longer that it would be required to obtain a good spectrum in this matrix in a separate experiment. Considering the thickness of the matrix, the thermal conductivity towards the outer end of the matrix cannot be ensured. Pure p-H_2 matrices are known to have excellent thermal conductivity, however, the presence of radical molecules and impurities of o-H_2 molecules are

known to affect it.[6,7] Therefore, slow evaporation or softening of the thick matrix during long periods of time cannot be excluded.

EPR spectra of 4 in argon and p-H_2 matrices

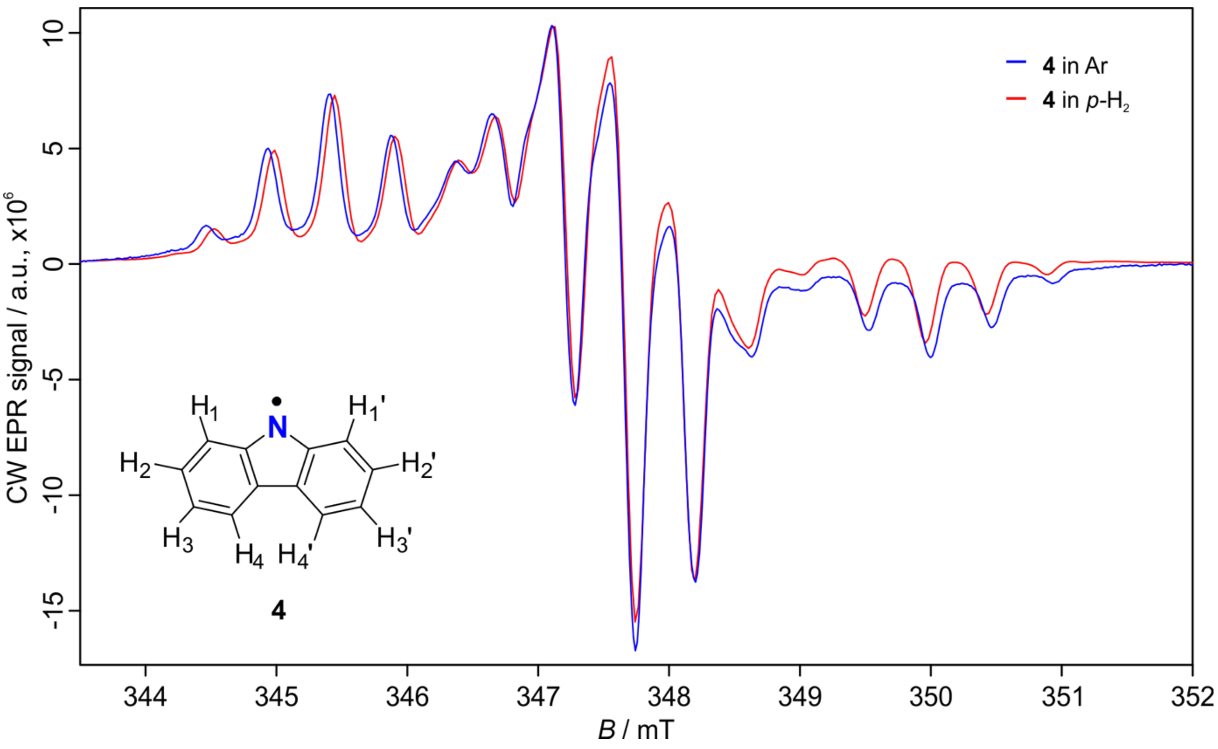

Figure S5. Matrix-isolated X-band EPR spectra of *N*-carbazolyl radical **4** in argon and p-H_2 matrices at cryogenic temperatures. The shown spectra are recorded at 40 dB, MA = 1 G and 10 scans in argon and 45 dB, MA = 0.5 G and 10 scans in p-H_2. The spectrum in p-H_2 was decreased in intensity by a factor of 6 to match the intensity of the spectrum in argon. The parameters were tested in the 40–50 dB range and MA was ranged between 0.25 and 1 G but the same resolution spectrum (not suffering from saturation) was obtained in all cases with the absolute intensity affected by the parameters used. The spectra in Ar and p-H_2 are shifted due to slight differences in the setup that led to an offset between both spectra after applying the field calibration correction. The spectrum in p-H_2 was recorded in the same conditions than all other spectra reported in this manuscript and used as a reference. The spectrum in Ar is showing a 0.9% factorial deviation compared to that in p-H_2.

For comparison, the spectrum of the *N*-carbazolyl radical (**4**) was simulated with the parameters shown in Table S3 while keeping the Lorentzian broadening values used to simulate radical **2** both in Ar and p-H_2 matrices (Figure S6). Since the spectra of **4** in argon and p-H_2 look very similar, the spectrum in p-H_2 was taken as the experimental reference. The simulation was running much slower than that of radical **2** with all 9 nuclei involved due to the higher spin of nitrogen compared to phosphorus. Therefore, the pair of hydrogen atoms with the lowest hfcc (H_4, A_{iso} was predicted to be 1.5 MHz for this pair of nuclei) was not included in the simulation. Nevertheless, the simulation with 0.055 mT broadening reproduces well all the features observed in the experimental spectrum, although that at 0.070 mT appears more accurate (Figure S6). Comparison of the spectra obtained in p-H_2 suggest that similar broadening

parameters can be used to simulate the spectra of both radicals **2** and **4**. However, the broadening required to simulate the spectrum of **4** in Ar differs from that of **2** (set to 0.15 mT for **2** in Ar, Figure S2). Independent of the differences in the broadening parameters, the difference in the hfcc between both radicals may be the cause for these inconsistencies in argon. The quintet signals can be clearly observed in the experimental spectrum of radical **4** in argon maybe due to their large isotropic hyperfine coupling constant (A_{iso}, 12.0 MHz) compared to those of radical **2** (in the order of 7.5 MHz). Additionally, the influence of the pair of hydrogens in position 3 for the *N*-carbazolyl radical was simulated to be 2.9 MHz and cannot be directly distinguished neither in the spectrum recorded in *p*-H_2 nor in argon matrix. Therefore, the data presented in this manuscript suggests that A_{iso} of 12 MHz can be distinguished in argon and *p*-H_2 matrices, whereas A_{iso} of roughly 8 MHz are not clearly observable in argon but can be well resolved in *p*-H_2. However, neither matrix can resolve an A_{iso} of approximately 3 MHz with the interference of larger interactions such as those observed with the nitrogen atom and hydrogens in positions 1 and 3.

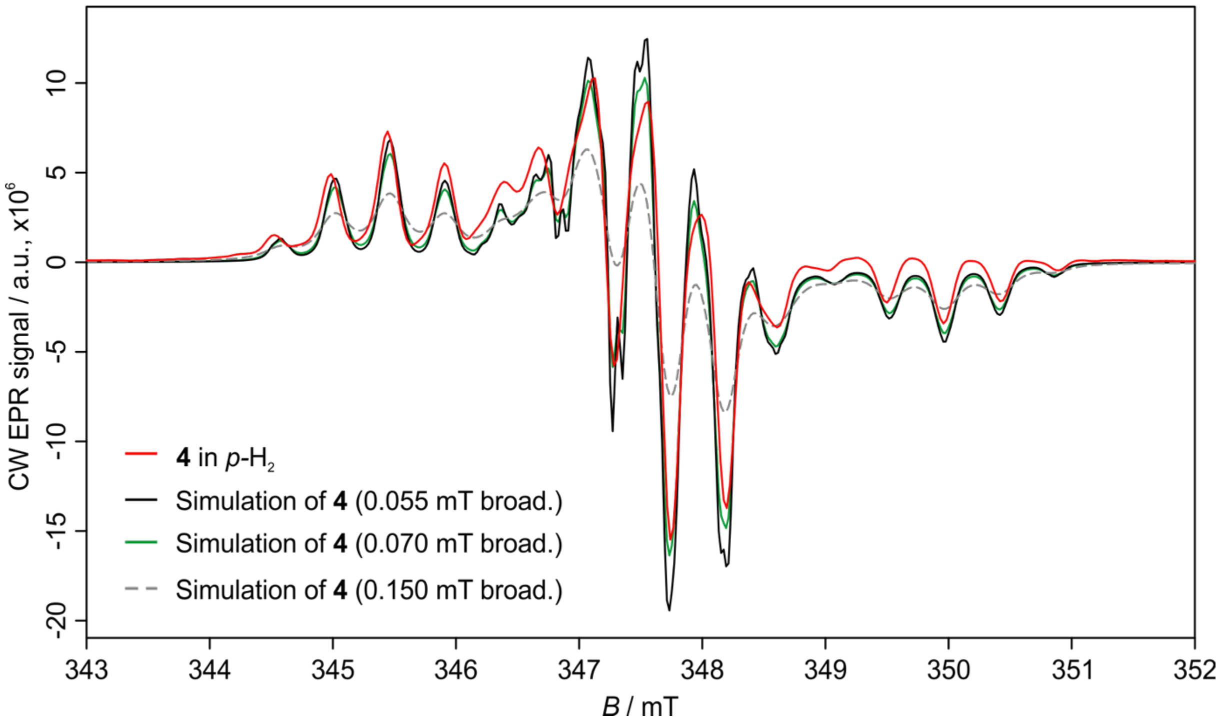

Figure S6. Matrix-isolated X-band EPR spectra of *N*-carbazolyl radical **4** in *p*-H_2 matrix (red trace, 45 dB, 0.5 G, 10 scans) and simulated spectra using the parameters in Table S3 with Lorentzian broadening parameters of 0.055 mT (black solid line), 0.070 mT (green solid line), and 0.150 mT (grey dashed line).

Table S3. Parameters utilized to simulate the spectrum of *N*-carbazolyl radical **4** in *p*-H_2 matrix**.** The hyperfine coupling constants (*A* or hfcc) are given in MHz and the broadening used was 0.055 mT. The hfcc of the protons are provided as their full 3x3 matrices with their signs kept from the calculations. The corresponding prime protons have the same parameters with their sign inverted in the out-of-diagonal terms. The sign of the hfccs has not been experimentally determined and has been kept as obtained from the ORCA calculation. The hfcc of the pair of hydrogens in position 4 could not be included in the simulation.

<table>
<tr><td colspan="5">N-carbazolyl radical 4
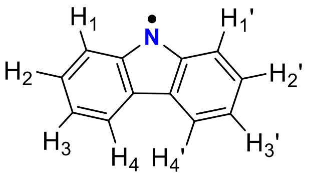
</td></tr>
<tr><td>Tensor</td><td>x</td><td>y</td><td>z</td><td>Isotropic</td></tr>
<tr><td>g</td><td>2.0019</td><td>2.0027</td><td>2.0042</td><td>2.0029</td></tr>
<tr><td>A(N)</td><td>−6</td><td>−6</td><td>63</td><td>15.3</td></tr>
<tr><td>A(H_1)</td><td colspan="3">$\begin{pmatrix} -13 & 0 & 0 \\ 0 & -9 & -4 \\ 0 & -4 & -14 \end{pmatrix}$</td><td>−12.0</td></tr>
<tr><td>A(H_2)</td><td colspan="3">$\begin{pmatrix} 1.8 & 0 & 0 \\ 0 & 3 & -1 \\ 0 & -1 & 4 \end{pmatrix}$ □</td><td>2.9</td></tr>
<tr><td>A(H_3)</td><td colspan="3">$\begin{pmatrix} -12 & 0 & 0 \\ 0 & -12 & 3 \\ 0 & 3 & -12 \end{pmatrix}$</td><td>−12.0</td></tr>
<tr><td>A(H_4)</td><td>–</td><td>–</td><td>–</td><td>–</td></tr>
</table>

Method of *o*/*p*-H_2 ratio determination

The infrared spectrum of solid *p*-H_2 is presented in Figure S7, showing a strong $S_1(0)$ absorption line at 4486.0 cm^{-1}. In addition, very strong double transition lines, $Q_1(0) + S_0(0)$ and $Q_1(1) + S_0(0)$, were observed at 4509.5 cm^{-1} and 4502.2 cm^{-1}, respectively, along with $S_1(0) + S_0(0)$ lines at 4835.5 cm^{-1} and 4843.7 cm^{-1} in the *p*-H_2 matrix. Additionally, broad bands corresponding to $Q_R(0)$ and $S_R(0)$ were observed at 4555.4 cm^{-1} and 4228.4 cm^{-1}, respectively. The double transition lines are relatively independent of crystal structure and can be used to determine the thickness of the *p*-H_2 matrix. Furthermore, the percentage of *o*-H_2 impurity can be estimated from the matrix thickness in combination with the area of the transition at 4228.4 cm^{-1}.

The method for estimating the percentage of *o*-H_2 in *p*-H_2 is well documented in the literature and is only briefly described here.[8,9] Three methods were used to estimate the matrix thickness, and the average value was taken: (a) Multiplying by a factor of 1.11 the difference between the intensities at 4229.4 cm^{-1} and 4100.0 cm^{-1}; (b) Integrating the 4509.4 cm^{-1} and 4502.2 cm^{-1} bands and dividing by 90; (c) Integrating the 4835.4 cm^{-1} and 4843.6 cm^{-1} bands and dividing by 7.

The resulting thickness is then used to calculate the percentage of *o*-H_2 impurity using the following formula: $f_{(o)} = \frac{\int Q1(0)dv}{d*35}$

where $Q_1(0)$ corresponds to the *o*-H_2 absorption at 4152.8 cm^{-1}, and 35 is the proportionality constant for the *o*-H_2 fraction.

The purity of *p*-H_2 has been measured by IR spectroscopy. Given that the experiments presented in this manuscript have been measured by EPR spectroscopy, the purity of *p*-H_2 could not be estimated for each experiment. Therefore, a purity of 99.2% of *p*-H_2 was estimated for all the experiments reported in this study.

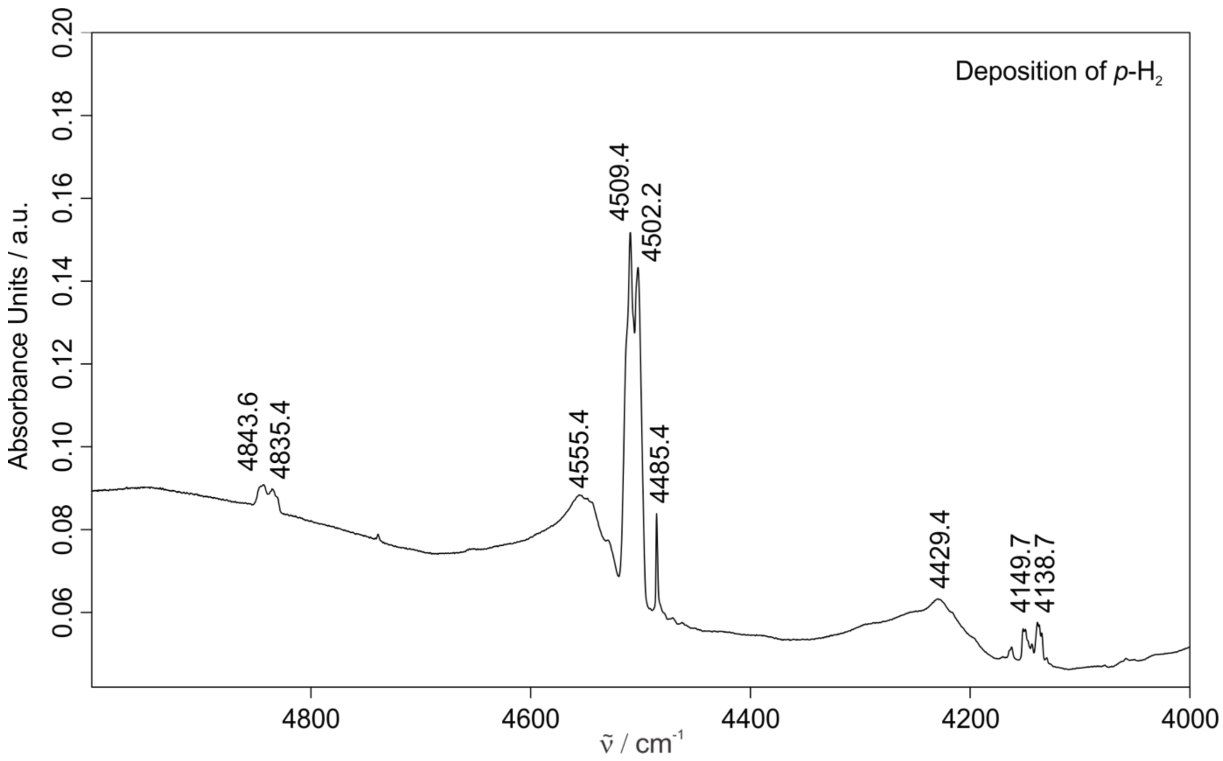

Figure S7. IR spectrum showing the deposition of *p*-H_2 to determine its purity.

Table S4. Estimation of the percentage of *o*-H_2 in *p*-H_2 and corresponding parameters, as mentioned in the text above for the EPR matrix isolation experiments in *p*-H_2.

**Experiment**	**2** in H_2
$Q_1(0)$	0.001
$Q_1 + S_0(0)$	1.200
$S_1(0) + S_0(0)$	0.089
A_{10} (4229.4 cm^{-1})	0.063
A_{10} (4100.0 cm^{-1})	0.047
d [Q_1+S_0(0)] (cm)	0.013
d [S_1(0) + S_0(0)] (cm)	0.013
d [ΔA_{10}[Q_R (0)] (cm)	0.018
% *o*-H_2 [Q_1+S_0(0)]	0.857 ± 0.019
% *o*-H_2 [S_1(0) +S_0(0)]	0.899 ± 0.026
% *o*-H_2 [ΔA_{10} (Q_R(0)]	0.628 ± 0.021
% *o*-H_2 Average	0.795

Methods

2,2,6,6-Tetramethylpiperidinyloxyl (**TEMPO**, **1**) was obtained from Sigma Aldrich (99%, sublimed) and used without further purification. **TEMPO** was handled under inert atmosphere to avoid its hydration from atmospheric water.

The synthesis and spectroscopic characterization of 5-iodo-5H-dibenzophosphole **3** can be found elsewhere.[10]

Low temperature matrices were generated by sublimation of the radical/precursor and co-deposition with excess gas: Ar (ALPHAGAZ 1, Ar ≥ 99.999 mol%, Air Liquide), *n*-H_2 (99.999%, Air Liquide) and *p*-H_2 (99.2%, prepared as described above), *n*-D_2 (99.96% atom %D, Sigma Aldrich) and Ne (>99.999%, Air Liquide). The mixture was deposited onto a copper rod at 2.5 K. *p*-H_2 was prepared using a home-build converter by exposing normal H_2 to porous iron(III) oxide (Sigma-Aldrich, hydrated, catalyst grade, 30−50 mesh) at 13.9 K for 1.5 h, following the procedure outlined in earlier studies.[9] EPR spectra was obtained at 2.5 K (and 5.5 K for the argon spectrum in Figure S5) with the EPR matrix setup discussed above using an Elexsys E500 CW-EPR spectrometer from Bruker. EPR spectra were recorded at frequencies around 9.7–9.8 GHz with modulating the field at 100 kHz and with the receiver gain set at 60 dB. The attenuation and modulation amplitude values were optimized for each measurement to avoid saturation and are indicated in the figure or figure caption. The attenuation value is provided instead of the microwave power for comfort in the manuscript and the correspondence to microwave power for the values used in this study are the following: 45 dB = 0.0065 mW; 50 dB = 0.0020 mW; 59 dB = 0.00026 mW; 60 dB = 0.00020 mW. The errors in the parameters directly extracted from the spectra are considered to be ± half the distance between points given that the spectrum was analyzed as a continuum line. That is 0.125 G (or 0.35 MHz) for the spectra of **TEMPO** and 0.083 G (rounded to 0.2 MHz) for the spectra of radical **2**. All spectra were tested for saturation and measured in the linear region. The field values were corrected with respect to a reference sample of Mn^{2+} in ZnS (a factorial offset of 0.9% was observed in the argon spectrum in Figure S5). The spin density calculations were performed using ORCA[11] at the UB3LYP/def2-TZVP[12] level of theory using the D3 dispersion correction[13]. The spin density was depicted using the IboView program with a resolution of 65,000.[14]

Optimized geometries

Table S5. Cartesian coordinates of the optimized structure of radical **2** calculated at the UB3LYP-D3/def2-TZVP level of theory (energies given in Hartrees).

Atomic Symbol	x	y	z
C	0.000001	2.962650	-1.437480
C	0.000001	3.493828	-0.148615
C	-0.000000	2.649433	0.954094
C	-0.000000	1.266677	0.766386
C	-0.000000	0.730671	-0.544465
C	0.000001	1.584806	-1.638446
H	0.000001	3.627448	-2.291784
H	-0.000001	4.566946	-0.007858
H	-0.000000	3.062788	1.955321
H	0.000001	1.188217	-2.646066
C	-0.000001	-1.266677	0.766386
C	-0.000000	-2.649433	0.954094
C	0.000001	-3.493828	-0.148615
C	-0.000001	-2.962650	-1.437480
C	-0.000001	-1.584806	-1.638446
C	-0.000001	-0.730671	-0.544465
H	0.000001	-3.062788	1.955321
H	0.000002	-4.566946	-0.007859
H	-0.000001	-3.627448	-2.291784
H	-0.000002	-1.188217	-2.646066
P	-0.000001	0.000000	2.036575
E = -803.33374563 ZPVE = 0.16020221			

References

[1] I. R. Dunkin, *Matrix-isolation Techniques: A Practical Approach*, Oxford University Press, New York, **1998**, https://doi.org/10.1093/oso/9780198558637.001.0001.

[2] E. Etienne, A. Pierro, K. C. Tamburrini, Bonucci, Alessio, E. Mileo, M. Martinho, V. Belle, *Molecules* **2023**, *28*, 1348, https://doi.org/10.1093/oso/9780198558637.001.0001.

[3] A. Savitsky, M. Plato, K. Möbius, *Appl. Magn. Reson.* **2010**, *37*, 415–434 https://doi.org/10.1007/s00723-009-0064-9.

[4] R. Owenius, M. Engström, M. Lindgren, M. Huber, *J. Phys. Chem. A* **2001**, *105*, 10967–10977, https://doi.org/10.1021/jp0116914.

[5] M. Plato, H.-J. Steinhoff, C. Wegener, J. T. Törring, A. Savitsky, K. Möbius, *Mol. Phys.* **2002**, *100*, 3711–3721, https://doi.org/10.1080/00268970210166246.

[6] M. Tsuge, Y.-P. Lee in *Mol. Laser Spectrosc.* (Eds.: V.P. Gupta, Y. Ozaki), Elsevier, **2020**, pp. 167–215, https://doi.org/10.1016/B978-0-12-818870-5.00005-8.

[7] F. M. Mutunga, K. M. Olenyik, A. I. Strom, D. T. Anderson, *J. Chem. Phys.* **2021**, *154*, 014302, https://doi.org/10.1063/5.0028853.

[8] M. E. Fajardo in *Phys. Chem. Low Temp.* (Ed.: L. Khriachtchev), Pan Stanford Publishing, Singapore, **2011**, https://doi.org/10.1201/9780429066276-6.

[9] K. Sundararajan, K. Sankaran, N. Ramanathan, R. Gopi, *J. Mol. Struct.* **2016**, *1117*, 181–191, https://doi.org/10.1016/j.molstruc.2016.03.068.

[10] M. Saraswat, A. Portela-González, K. Wulff, A. K. Eckhardt, *J. Org. Chem.* **2025**, *90*, 10616–10624, https://doi.org/10.1021/acs.joc.5c00840.

[11] F. Neese, *WIREs Comput. Mol. Sci.* **2022**, *12*, e1606, https://doi.org/10.1002/wcms.1606.

[12] F. Weigend, R. Ahlrichs, *Phys. Chem. Chem. Phys.* **2005**, *7*, 3297–3305, https://doi.org/10.1039/b508541a.

[13] S. Grimme, S. Ehrlich, L. Goerigk, *J. Comput. Chem.* **2011**, *32*, 1456–1465, https://doi.org/10.1002/jcc.21759.

[14] G. Knizia, J. E. M. N. Klein, *Angew. Chem. Int. Ed.* **2015**, *54*, 5518–5522, https://doi.org/10.1080/00268970210166246
